# Protective effect of butin against ischemia/reperfusion-induced myocardial injury in diabetic mice: involvement of the AMPK/GSK-3β/Nrf2 signaling pathway

**DOI:** 10.1038/srep41491

**Published:** 2017-01-27

**Authors:** Jialin Duan, Yue Guan, Fei Mu, Chao Guo, Enhu Zhang, Ying Yin, Guo Wei, Yanrong Zhu, Jia Cui, Jinyi Cao, Yan Weng, Yanhua Wang, Miaomiao Xi, Aidong Wen

**Affiliations:** 1 Department of Pharmacy, Xijing Hospital, Fourth Military Medical University, Xi’an, 710032, China; 2 College of Pharmacy, Shaanxi University of Chinese Medicine, XianYang 712083, PR China

## Abstract

Hyperglycemia-induced reactive oxygen species (ROS) generation contributes to development of diabetic cardiomyopathy (DCM). This study was designed to determine the effect of an antioxidant butin (BUT) on ischemia/reperfusion-induced myocardial injury in diabetic mice. Myocardial ischemia/reperfusion (MI/R) was induced in C57/BL6J diabetes mice. Infarct size and cardiac function were detected. For *in vitro* study, H9c2 cells were used. To clarify the mechanisms, proteases inhibitors or siRNA were used. Proteins levels were investigated by Western blotting. In diabetes MI/R model, BUT significantly alleviated myocardial infarction and improved heart function, together with prevented diabetes-induced cardiac oxidative damage. The expression of Nrf2, AMPK, AKT and GSK-3β were significantly increased by BUT. Furthermore, in cultured H9c2 cardiac cells silencing Nrf2 gene with its siRNA abolished the BUT’s prevention of I/R-induced myocardial injury. Inhibition of AMPK and AKT signaling by relative inhibitor or specific siRNA decreased the level of BUT-induced Nrf2 expression, and diminished the protective effects of BUT. The interplay relationship between GSK-3β and Nrf2 was also verified with relative overexpression and inhibitors. Our findings indicated that BUT protected against I/R-induced ROS-mediated apoptosis by upregulating the AMPK/Akt/GSK-3β pathway, which further activated Nrf2-regulated antioxidant enzymes in diabetic cardiomyocytes exposed to I/R.

The incidence and prevalence of diabetes mellitus (DM) is growing rapidly from 135 million in 1995 to an estimated 330–380 million in 2025^1^. The World Health Organization has projected that DM related deathrate will be doubled between 2005 and 2030, of which type 2 diabetes mellitus (T2DM) will account for over 90% (http://www.who.int/diabetes/en/). The proportion of cardiovascular disease morbidity and mortality caused by DM has increased over the past 50 years according to the Framingham heart study implying that more efforts are needed to optimize the control of cardiovascular disease risk factors among individuals with DM^2^. However, up to now, there is no effective specific treatment available for diabetic cardiomyopathy (DCM).

Several mechanisms are likely to contribute to the increased cardiovascular disease risk noted in patients with DM. Hyperglycemia seen as a result of diabetes causes early maladaptation in cardiac metabolism^3^. Sustained hyperglycemia increases the production of reactive oxygen species (ROS), with altering the cellular redox status, antioxidant mechanisms and membrane function, followed by contractile dysfunction within weeks in the diabetic heart^4^. Four major important enzyme systems for ROS production are xanthine oxidase, nicotinamide adenine dinucleotide phosphate (NADPH) oxidase, a dysfunctional endothelial nitric oxide synthase (eNOS) and the enzymes of the mitochondrial respiratory chain^1^. Under physiological conditions, ROS production via NADPH oxidase is eliminated efficiently by antioxidants, while excess activation of NADPH oxidase disturbs the balance and leads to oxidative stress, mitochondrial dysfunction, and impaired antioxidant gene expression. Thus, improving antioxidant enzyme activities and suppressing oxidative stress, are appropriate targets in treating diabetes-induced cardiovascular disease. There is a wide variety of factors associated with the cellular response to oxidative stress, and the NF-E2-related factor (Nrf2) pathway is regarded as the most important factors^5^. Nrf2 is a nuclear transcription factor that binds to antioxidant-response element (ARE) and regulates expression and coordinated induction of a battery of chemoprotective genes in response to antioxidants, oxidants, and radiations, including NAD (P) H: quinine oxidoreductase 1 (NQO1), NRH: quinone oxidoreductase 2 (NQO2), glutathione S-transferase Ya subunit (GST Ya Subunit), heme oxygenase 1 (HO-1), and γ-glutamylcysteine synthetase (γ-GCS), also known as glutamate cysteine ligase (GCL)^6^. INrf2 (inhibitor of Nrf2) or Keap1 retains Nrf2 in the cytoplasm, once upon exposure of cells to oxidative stress or electrophilic compounds, Nrf2 is free from Keap1 and translocates into the nucleus where it up-regulates the expression of numerous cytoprotective phase II detoxifying enzymes and antioxidant genes^7^.

Glycogen synthase kinase-3 (GSK-3) is a ubiquitously expressed serine/threonine kinase that has versatile biological functions in cells, including regulation of metabolism, cell growth/death, and gene transcription^8^. Although both GSK-3α and GSK-3β appear to be equally important in certain aspects, GSK-3α has a more critical role in regulating hepatic glucose metabolism and insulin sensitivity, and GSK-3β is the predominant regulator of glycogen synthase, Wnt signaling and sensitization to apoptosis^9^. Contrary to most signalling kinases, GSK-3β is active in unstimulated cells and sensitizes cells to death-promoting insults. In heart, GSK-3 has several important roles. Recently, inhibition of GSK-3β during ischemia and reperfusion (I/R) has been implicated as a cardioprotective mechanism^10^. However, the underlying mechanisms of cardioprotection afforded by GSK-3β in DCM remain largely unknown.

Traditional Chinese medicine (TCM) performs a good clinical practice and is showing a bright future in the treatment of DM. TCM treatment has certain advantages of less toxicity and/or side effects, and provides multiple therapeutic effects^11^. Flavonoids, a group of naturally occurring polyphenolic compounds, has been traditionally used in the drugs for the prevention and therapy of free radical-mediated human diseases, such as inflammation, neuronal degeneration, atherosclerosis, ischemia, and many cardiovascular diseases^12^. Butin (BUT, 7, 30, 40-trihydroxydihydroflavone, Fig. 1), a plant dietary flavonoid, is one of the major biologically active components of the heartwood of Dalbergia odorifera (DO). It has been reported to possess biological properties such as anti-implantation and skin-whitening activity^13^. A BUT-rich extract obtained from DO is always taken to treat ischemia, blood stasis, inflammation and necrosis in China and Korea^14^. It was also reported that BUT provided protective effects against H2O2-induced cell injury by scavenging ROS and activating antioxidant enzymes, and activated of PI3K/Akt/OGG1 pathway to protected against oxidative DNA damage^15^,^16^. Since little is known about the effects of BUT on GSK-3β and the relationship between GSK-3β and Nrf2 in DCM. We performed this study to determine whether BUT protected against experimental diabetic cardiomyocytes which exposed to I/R *in vitro* and *in vivo* via AMPK/Akt/GSK-3β/Nrf2 signaling pathway.

## Results

### BUT inhibited myocardial injury following myocardial I/R in diabetic mice

To investigate whether BUT has cardioprotective effects against I/R injury in diabetic mice, cardiac function after I/R injury in diabetic mice was examined. The degrees of cardiac function disorder in DM + I/R group were more serious in I/R group. Compared to the DM + sham group, I/R injury caused significant decrease of +LV dP/dt _max_ (Fig. 2A), LV dP/dt _min_ (Fig. 2B) and LVDP (Fig. 2C) in diabetic mice. In BUT and MET treatment group, LVDP, +LV dP/dt max and LV dP/dt_min_ were improved significantly at the end of reperfusion when compared with DM + I/R group. These results indicated that BUT improved cardiac functional recovery in diabetic mice subjected to I/R.

**Figure 1:**
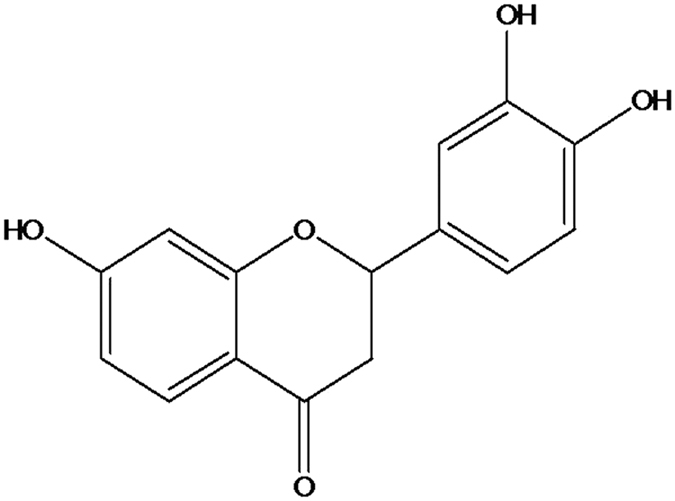
Chemical structure of butin.

**Figure 2:**
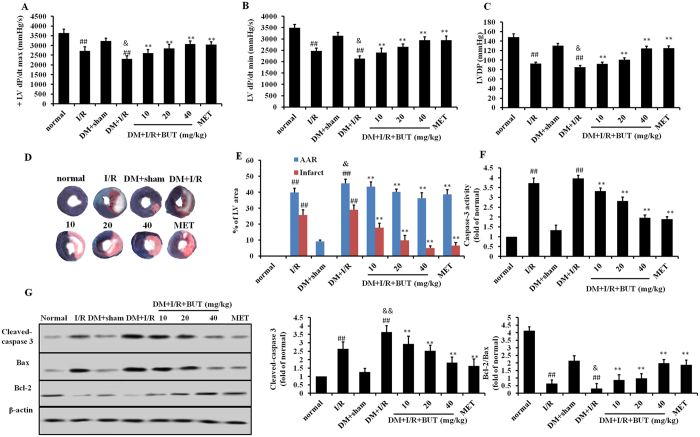
Cardioprotective of BUT in diabetic mice with I/R injury. BUT or metformin (MET) were administered by gavage every other day for 15 days after induction of diabetes. +LV dP/dt max (**A**), LV dP/dt_min_ (**B**) and LVDP (**C**) were measured after 30 minutes ischemia and 6 hours reperfusion. LVEDP, left ventricular end diastolic pressure; LVdP/dtmax, the instantaneous first derivation of left ventricle pressure. (**D**) Representative images of infarct size as stained by Evans Blue and TTC. (**E**) Myocardial infarct size and area at risk. AAR, area at risk; LV, left ventricle. (**E**) Myocardial caspase-3 activity. (**F**) Cleaved-caspase 3, Bax and Bcl-2 were measured in heart tissues. Values (n = 6–8 per group) are expressed as means ± SD. ^##^*P* < 0.01 vs normal or DM + sham group, **P < 0.01 vs DM + I/R group, ^&^*P* < 0.05 vs I/R group.

As myocardial function improved by treatment with BUT, we measured the infarct size and area at risk (AAR). The AAR in DM + I/R group were larger than that in I/R group, and BUT could significantly reduce AAR at 40 mg/kg. As shown in Fig. 2D, no myocardial infarction was observed in normal-group hearts. In DM + sham group, DM caused increasing of myocardial ischemic area, but there was no infarction. I/R resulted in significant infarction in DM + I/R group mice compared to DM + sham (49.6% ± 3.1% versus DM + sham, *P* < 0.01). BUT significantly reduced the infarct size (14.5% ± 5.1%) compared to DM + I/R. The relationship between the size of the risk zone and the size of the necrotic zone was plotted (Fig. 1) and analyzed using analysis of covariance (Supplementary Table 2). The results showed that there was no significant effect on the relationship between AAR and infarct.

Apoptosis is the major mechanism of cell death immediately following I/R. Myocardial caspase-3 activity and Bax/Bcl-2 are two very specific indicators of cardiomyocyte apoptosis. In this study, caspase-3 activity in serum and heart tissue and Bax/Bcl-2 ratio were measured. As expected, caspase-3 levels were minimally detected in the sham-group, whereas MI/R significantly increased caspase-3 levels. Pretreatment significantly reduced caspase-3 levels in a dose dependent manner (Fig. 2E). Consistent with caspase-3 results in serum, cleaved-caspase-3 expression and Bax/Bcl-2 ratio were significantly increased in the MI/R group. (Figure 2F). And these indicators substantially were reduced in BUT and MET group compared to those in MI/R group, with statistic significance (*P* < 0.01).

Cardiomyocyte necrosis is characterized by cellular content release. To determine whether BUT attenuated MI/R-induced cardiomyocyte necrosis, plasma CK-MB and LDH levels were measured after reperfusion. Plasma CK-MB and LDH levels were increased by I/R significantly and BUT treatment markedly decreased LDH and CK-MB (Supplementary Material, Figure S2A and B) levels in dose dependent manner. These indicators supported that BUT decreased myocardial necrosis post-MI/R in diabetic mice. Together, these data suggested BUT decreased MI/R myocardial injury in diabetic mice.

### BUT enhances antioxidant capacity in cardiac tissues

**Figure 3:**
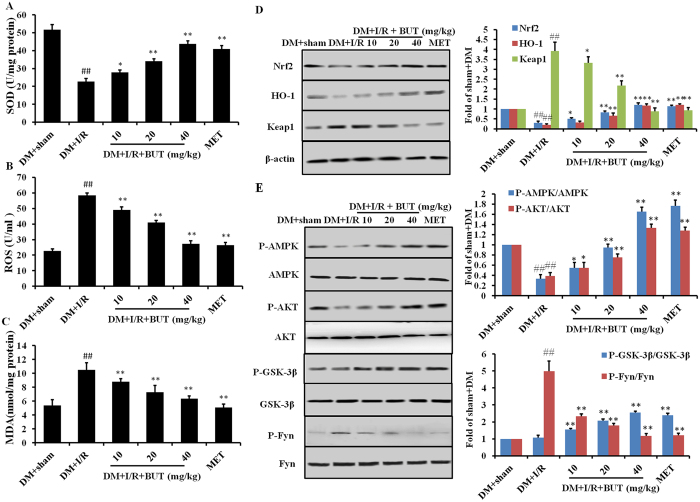
BUT up-regulated the expression of SOD and attenuated MDA and ROS levels in the heart. Diabetes mice received I/R treatment in the presence or absence of BUT pre-treatment, the expression level of SOD (**A**), ROS (**B**) and MDA (**C**) in the heart were measured as described in Materials and methods. Effects of BUT on AMPK, Akt and GSK3β phosphorylation and Nrf2 expression in heart treated with I/R. Immunoblotting of protein extracts from heart of diabetic mice treated with BUT or MET. (**D**) Expression of Nrf2, keap1 and HO-1 in the heart of diabetic mice with or without BUT treatment. (**E**) Phosphorylation of AMPK, Akt, GSK3β and Fyn in the hearts of diabetic mice with or without BUT treatment. The columns and errors bars represent means ± SD. ^##^*P* < 0.01 vs DM + sham group, **P < 0.01 vs DM + I/R group.

To test if the protective effects of BUT against I/R-induced heart dysfunction were due to its antioxidative activity, we measured the oxidative stress associated parameters in the heart. As shown in Fig. 3 and Figure S2, we found that diabetes mice treated with I/R showed significant (*P* < 0.05) decrease in the activities of GSH-Px, GSH, SOD, CAT and GR, in the same time, MDA production in the heart was increased compared with DM + sham group. Pretreatment with BUT (10, 20 and 40 mg/kg) or MET (50 mg/kg) before the I/R treatment could reverse these effects, which significantly included up-regulation of these enzymes activities and attenuated MDA levels in the heart compared to DM + I/R group (Fig. 3B). BUT pretreatment also significantly decreased the intracellular levels of ROS in a dose-dependent manner (Fig. 3C). These findings suggested that BUT could considerably improve cellular antioxidative defense capacity against oxidative stress.

Above results showed that BUT could protect cardiac oxidative damage of I/R induction though inhibition ROS production and promotion of antioxidative proteins. As we known, GSH-Px, GSH, SOD and CAT are downstream of Nrf2, therefore, whether BUT protected the heart from I/R by activating Nrf2 was examined by measuring Nrf2 expression and its transcription function in diabetic heart. From Western blot analysis (Fig. 3D), Nrf2 expression was found to be significantly decreased after I/R in diabetic heart and BUT treatment resulted in a dose-dependent increase of it, as well as the expression levels of HO-1. The expression levels of Keap1, the repressor of Nrf2 which leads to ubiquitination and degradation of Nrf2, were significantly attenuated in diabetic mice heart treated with BUT.

To investigate whether AMPK, Akt and GSK-3β might be responsible for the activation of Nrf2 and protective effect of BUT, protein kinase activities in diabetic heart were assessed by determining the phosphorylated forms of AMPK, Akt and GSK-3β. GSK-3β acts as upstream of Fyn Kinase, which regulates the nuclear export and degradation of Nrf2. In our study, diabetic C57BL/6J mice were subjected to I/R to examine the functional status of GSK-3β during ischemia and reperfusion. As the results shown I/R slightly increased phosphorylation of GSK-3β in the diabetic mouse heart (Fig. 3E). Pretreatment with BUT significantly increased the phosphorylation of GSK-3β (P-GSK-3β/GSK-3β ratios, *P* < 0.01). We also found that Fyn was significantly increased by I/R, and BUT decreased phosphorylation of Fyn in a dose dependent manner. Moreover, BUT treatment increased the phosphorylation of Akt and AMPK in diabetic hearts. We also found that BUT the same pathway in non-diabetic mice subjected to I/R (Supplementary Material, Figure S3). These results indicated that treated with BUT facilitated the expression of Nrf2 by inducing ARE-mediated expression of antioxidant genes, and the possible mechanism was through phosphorylation of AMPK/Akt/GSK-3β pathway.

### BUT enhanced the expression of Nrf2 and its downstream antioxidant enzyme in H9c2 cells

**Figure 4:**
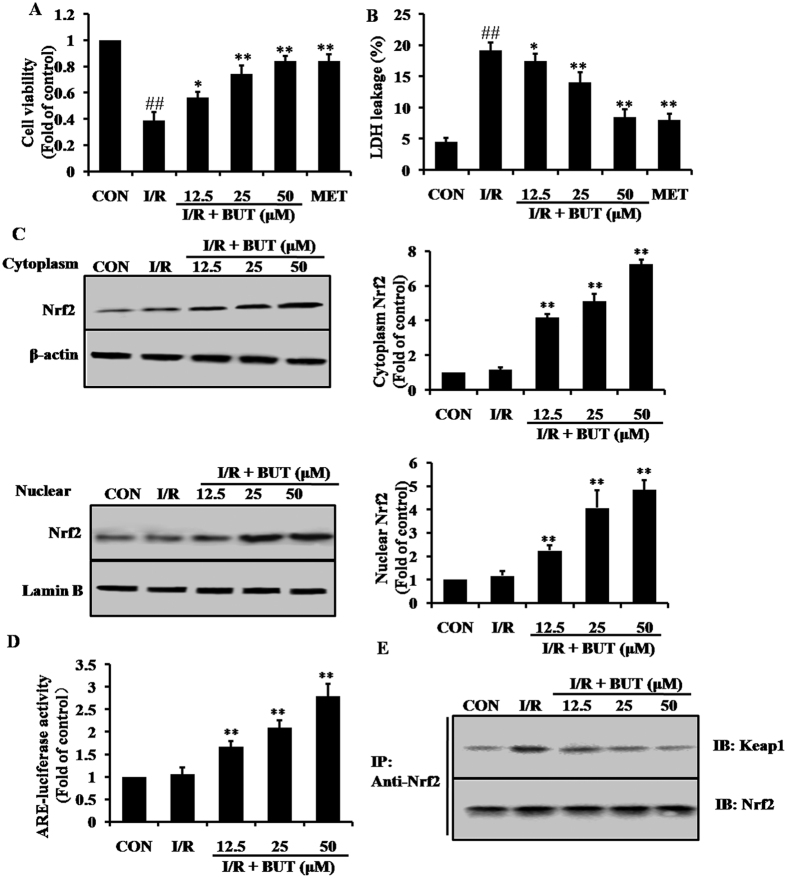
Effects of BUT on expression of Nrf2 and its downstream antioxidant enzyme in H9c2 cells. (**A**) Cells were pretreated by BUT (12.5, 25 and 50 μM) or MET (40 μM) for 6 h, and then subjected to I/R. Cell viabilities were assayed by MTT. The data are shown as fold of control. (**B**) LDH assay in cells administered with BUT (12.5, 25 and 50 μM) for 6 h prior to I/R. Cellular death determined as LDH leakage into medium. (**C**) Immunoblot assay showing that BUT induced the cytoplasm and nuclear levels of Nrf2 in a dose-dependent manner. (**D**) BUT increased the transcriptional activity of Nrf2 determined by the ARE luciferase reporter assay. (**E**) BUT disrupted the Keap1-Nrf2 complex. H9c2 cells were treated with BUT for the indicated times, subsequently, the cell lysates were prepared for immunoprecipitation (IP) and probed with anti-Keap1 antibody for immunoblot analysis (IB). The precipitates were also blotted with anti-Nrf2 antibody to ensure that equal amounts of Nrf2 were pulled down under each condition. The columns and errors bars represent means ± SD. ^##^*P* < 0.01 vs control group, **P < 0.01 vs I/R group.

The above findings clearly suggested the possible contribution of BUT to the protection against I/R-induced myocardial injury in diabetic heart through up-regulated AMPK, Akt, Nrf2 and GSK-3β expression. To further explore the specific mechanism, H9c2 cells were pretreated with BUT for 6 h and then subjected to I/R. The protective effect of BUT against I/R-induced cytotoxicity was detected by MTT assay and LDH leakage. As shown in Fig. 4A, BUT treatment exhibited a strong dose-dependent protective effect given pretreatment for 6 h. The survival rate increased from 38.7% (with I/R treatment alone) to 56.3 ± 4.16%, 74.3 ± 6.21%, and 84.3 ± 3.51% with 12.5, 25, and 50 μM BUT treatments, respectively. LDH leakage increased significantly in I/R group compared to that of control group (Fig. 4B, *P* < 0.01). BUT (12.5, 25 and 50 μM) markedly reduced I/R induced cell death by decreasing LDH leakage to 11% ± 1.7%, 10.3% ± 0.9%, and 7.3% ± 0.9% (*P* < 0.01). Together, these results indicated that BUT significantly preserved cell apoptosis and death induced by I/R injury in a dose-dependent manner.

To verify the results *in vivo*, we attempted to examine the expression level of Nrf2 proteins in BUT-stimulated H9c2 cells. The Nrf2 levels in cytoplasm and nuclear were increased by treatment with BUT in a dose-dependent manner (Fig. 4C). Results of cell immunofluorescence staining also indicated that BUT pretreatment increased Nrf2 accumulation in the nucleus and cytoplasm (Supplementary Material, Figure S4). In addition, BUT increased the transcriptional activity of Nrf2-ARE in a dose-dependent manner using the ARE-luciferase reporter assay (Fig. 4D). The effect of BUT on the Nrf2-Keap1 interaction was then examined by immunoprecipitation with anti-Nrf2 antibody, followed by detection of Keap1 immunoreactivity using immunoblotting (IB). We found that BUT disrupted the interaction of Nrf2 with Keap1 in a dose-dependent manner (Fig. 4E).

**Figure 5:**
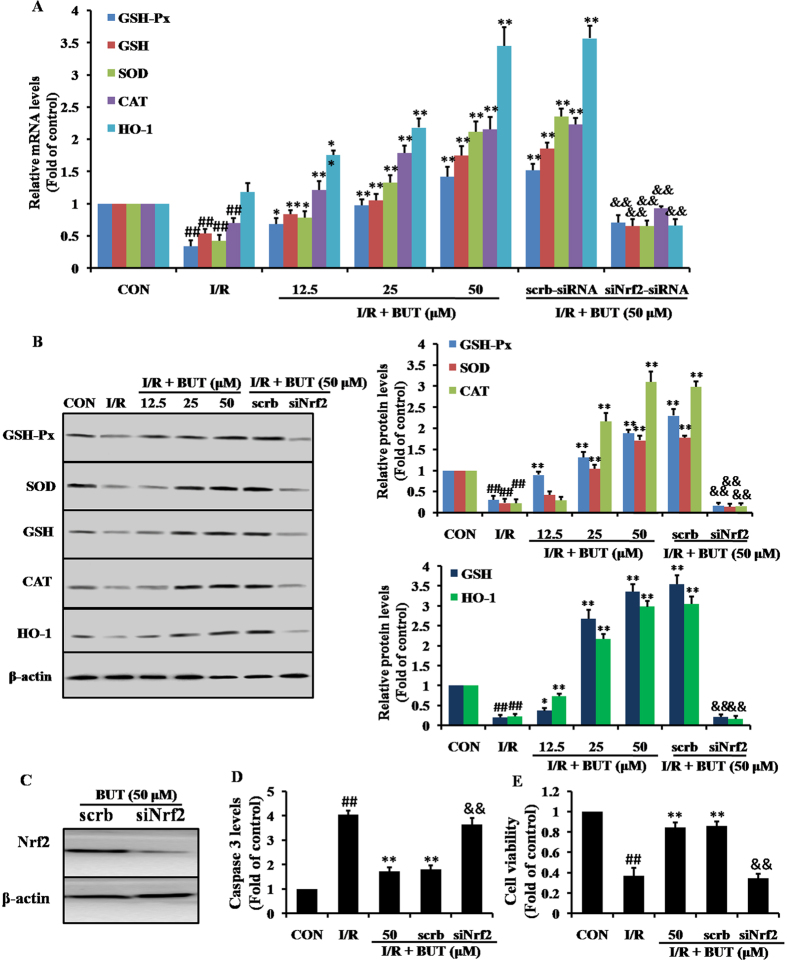
Effect of BUT on Nrf2-related proteins and the important role of Nrf2. (**A**) The mRNA levels of GSH-Px, GSH, SOD, CAT and HO-1 were measured by real-time PCR in different treatment groups. (**B**) Immunoblot and densitometry analysis showing the protein expression of GSH-Px, GSH, SOD, CAT and HO-1 in different treatment groups. (**C**) Immunoblot analysis showing the protein expression of Nrf2 in H9c2 cells treated with Nrf2 specific siRNA (40 nM). (**D**) Nrf2 specific siRNA exhibited markedly higher levels of caspase 3 (**E**) and significantly lower cell viability (**F**). The columns and errors bars represent means ± SD. **P < 0.01 vs I/R group. ^##^*P* < 0.01 vs control group. ^&&^*P* < 0.01 vs crambled control RNA.

Next, we investigated the effect of BUT-induced Nrf2 signaling by measuring the protein expression of five Nrf2-target downstream genes, namely GSH-Px, GSH, SOD, CAT and HO-1, at both mRNA (Fig. 5A) and protein level (Fig. 5B). Treatment with BUT significantly increased the expression of these antioxidant genes at both mRNA and protein levels while I/R treatment slightly increase HO-1, CAT mRNA and protein expression and significantly decreased SOD, GSH-Px and GSH mRNA and protein expression. However, all these antioxidant expression levels were higher in BUT pretreatment group than that these in I/R group.

To further confirm whether the suppression of I/R-induced cell apoptosis in H9c2 cells by BUT occurred in an Nrf2-dependent manner, we transfected H9c2 cells with Nrf2-specific siRNA and then subjected them to I/R. Results showed that after cells were exposed to siRNA for 48 h, the specific siRNA could almost completely silenced Nrf2 expression (Fig. 5C) as well as the expression of its downstream target genes at the protein and gene levels (Fig. 5A and B). Nrf2 specific siRNA also exhibited markedly higher levels of caspase 3 (Fig. 5E), and significantly lower cell viability (Fig. 5F) than cells that transfected with crambled control RNA transfection. The results indicated that Nrf2 played an essential role in the protective effect of BUT against I/R-induced apoptosis.

### BUT-induced Nrf2 upregulation is mediated by the Akt and AMPK pathway

**Figure 6:**
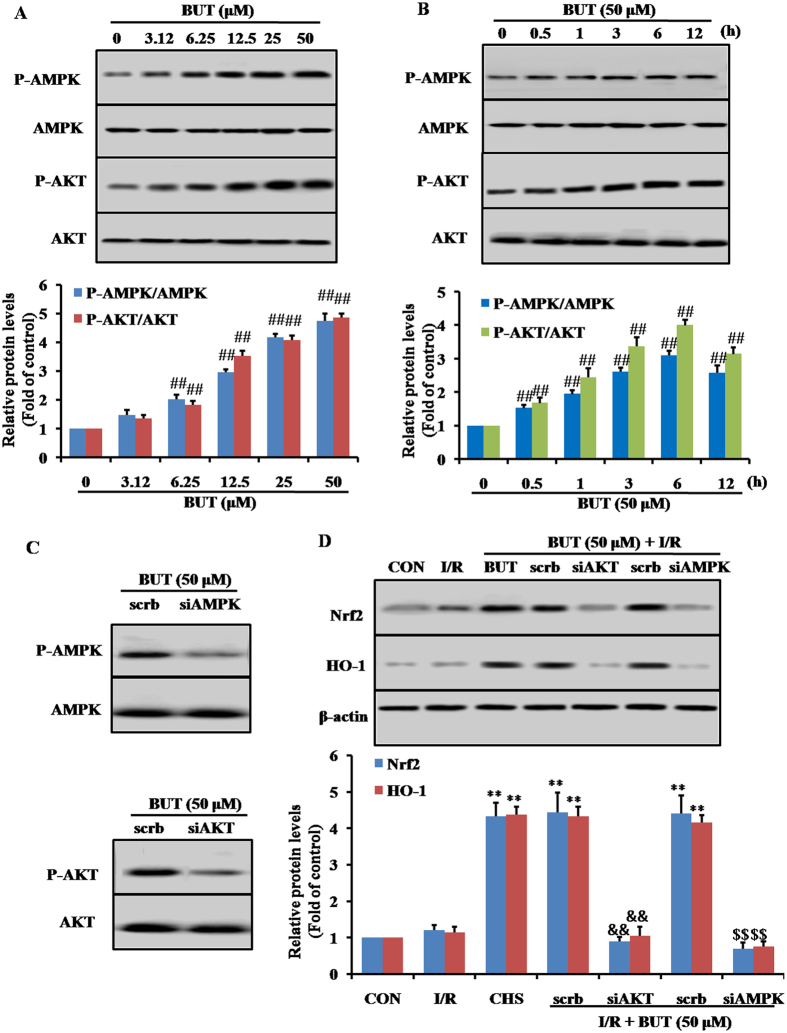
Antiapoptotic effect of BUT on I/R-induced H9c2 cell injury is mediated through Akt and AMPK-dependent activation of Nrf2. BUT treatment induced concentration- (**A**) and time-related (**B**) increases in the phosphorylation of AMPK and Akt in H9c2 cells. ^##^*P* < 0.01 vs 0 h or 0 μM group. H9c2 cells were treated with 50 μM BUT with or without Akt siRNA (30 μM) or AMPK siAMPK (30 μM), which was transfected into cells 48 h before I/R-exposure. The scramble represents the non-specific siRNA. The levels of AMPK, AKT (**C**) and Nrf2, HO-1 (**D**) were determined as indicated by Western blot assay. The columns and errors bars represent means ± SD. **P < 0.01 vs I/R group. ^##^*P* < 0.01 vs control group. ^&&^*P* < 0.01 and ^$$^*P* < 0.01 vs crambled control RNA.

Previous studies had shown that the Akt and AMPK pathways can up-regulate Nrf2-induced antioxidative proteins expression. To investigate the possible signal pathways involved in BUT-induced Nrf2 activation and antioxidative proteins expression, we examined the phosphorylation of these upstream kinases. Upon BUT treatment, concentration- (Fig. 6A) and time-related (Fig. 6B) increases in the phosphorylation of AMPK and Akt were observed in H9c2 cells. Next, we examined whether BUT enhanced Nrf2 expression via the Akt and AMPK pathways. Cells were transfected with Akt- and AMPK-specific siRNA, and then pretreated with BUT and subjected to I/R (Fig. 6C). The protein levels of Nrf2 and HO-1 were markedly lower in those siRNA treated cells than in cells in the BUT treated group (Fig. 6D). BUT mediated cytoprotection against I/R injury was also attenuated by siRNA (Supplementary Material, Figure S5A and B).

**Figure 7:**
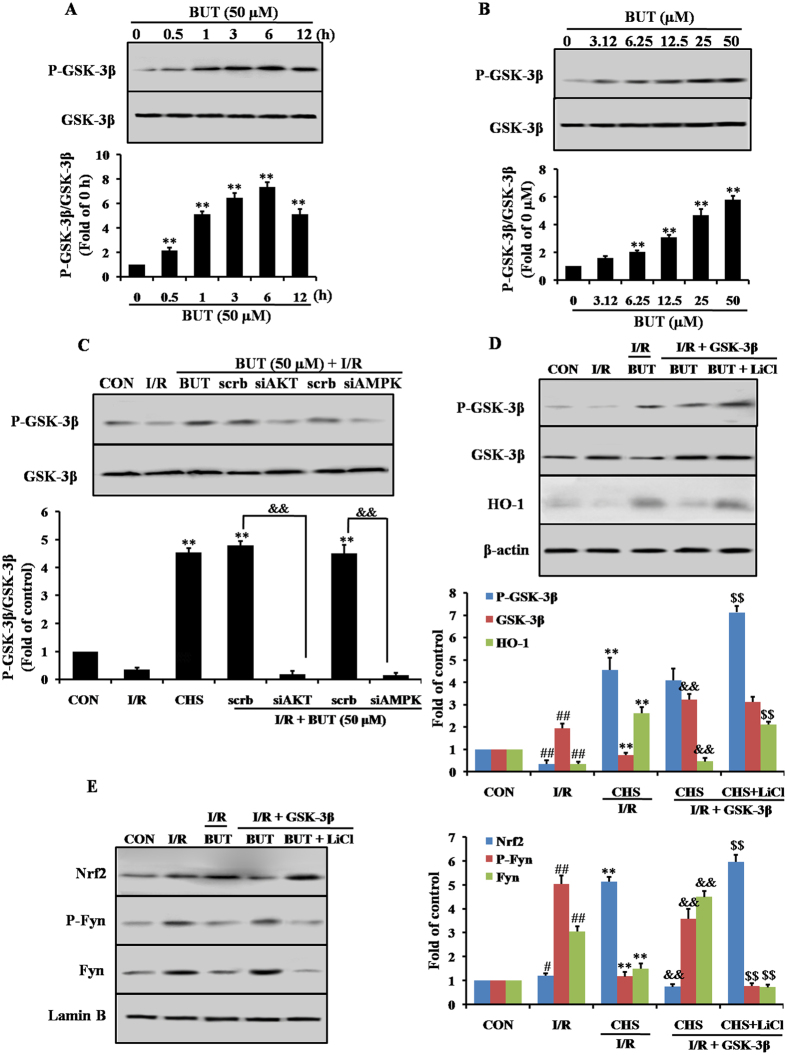
BUT-induced Nrf2 signaling was dependent on GSK-3β. BUT treatment induced concentration- (**A**) and time-related (**B**) increases in the phosphorylation of GSK-3β in H9c2 cells. **P < 0.01 vs 0 h or 0 μM group. (**C**) Effect of BUT induced GSK-3β expression was abolished by Akt or AMPK siAMPK. The columns and errors bars represent means ± SD. **P < 0.01 vs I/R group. ^##^*P* < 0.01 vs control group. ^&&^*P* < 0.01 vs crambled control RNA. H9c2 cells were transfected with the empty vector pcDNA3 or vectors encoding HA-tagged wild-type GSK3β (WT-GSK3β-HA). Upon transfection, H9c2 were exposed to different treatments as indicated. (**D**) Cytosolic fractions were extracted from various groups as indicated and underwent immunoblot analysis of GSK-3β, P-GSK-3β and HO-1. (**E**) Nuclear fractions were extracted from various groups as indicated and underwent immunoblot analysis of Nrf2, Fyn and P-Fyn. **P < 0.01 vs I/R group. ^##^*P* < 0.01 vs control group. ^&&^*P* < 0.01 vs BUT + I/R. ^$$^*P* < 0.01 vs BUT + I/R + GSK-3β.

Another downstream target of both Akt and AMPK is GSK-3β, and it is an upstream of Nrf2. In an effort to identify downstream of AMPK and Akt, we next examined the effect of AMPK and Akt on GSK-3β phosphorylation and its role in myocardial cells protection. BUT treatment increased the phosphorylation of GSK-3β at serine 9 residue in dose- and time-dependent manners (Fig. 7A and B), and this increase was prevented by Akt- and AMPK-specific siRNA treatment (Fig. 7C). To further study the role of GSK-3β in activating Nrf2 pathway, GSK-3β was over-expressed. Over expression of GSK-3β caused the increase of cytosolic GSK-3β expression under I/R conditions and p-GSK-3β-ser9 level did not go up correspondingly (Fig. 7D). Overexpressed GSK-3β went beyond the action of BUT so that p-GSK3β-ser9 level in transfected H9c2 subjected to I/R could not be raised further by BUT. However, GSK-3β antagonist LiCl reinforced phosphorylation of GSK-3β at ser9 in GSK-3β-transfected H9c2 cells. I/R-increased GSK-3β activity was associated with enhancement of nuclear accumulation of Fyn, Fyn phosphorylation and limited Nrf2 nuclear translocation (Fig. 7E). BUT counteracted these I/R elicited effects and expectedly amplified Nrf2 signaling pathway such as HO-1 up-regulation. Collectively, these results suggested that BUT activated AMPK and Akt induces phosphorylation of GSK-3β at Ser9, which may inhibit GSK-3β-mediated Nrf2 degradation, resulting in Nrf2 nuclear accumulation and activation of Nrf2 related proteins in myocardial cells.

### PI3K/Akt pathway is necessary for BUT-induced AMPK-mediated GSK-3β phosphorylation

**Figure 8:**
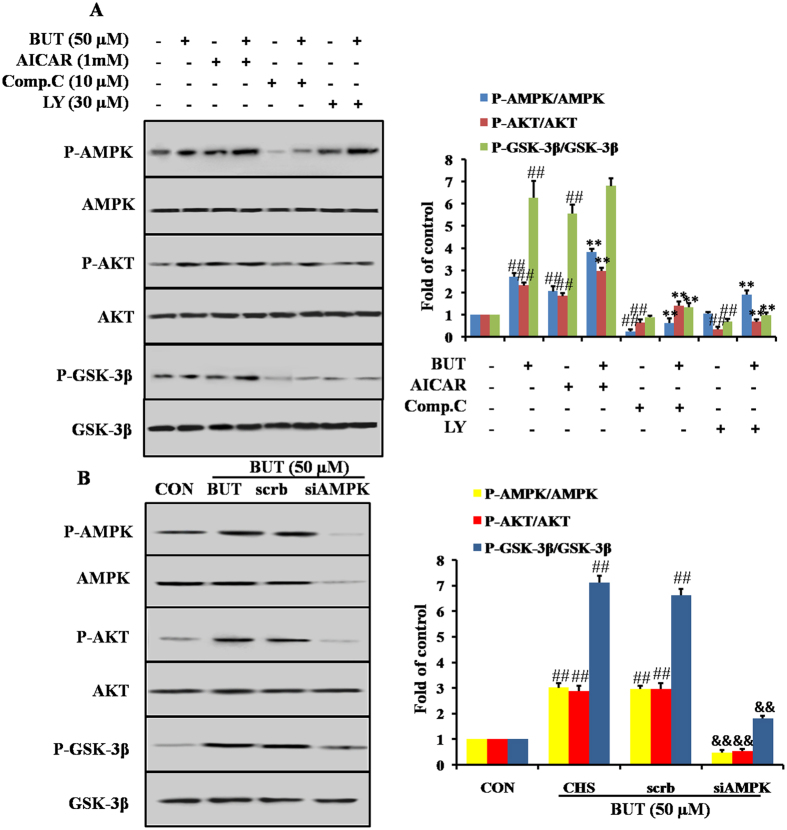
PI3K/Akt pathway was necessary for BUT-induced AMPK-mediated GSK-3β phosphorylation. (**A**) H9c2 cells were treated with 10 μM Compound C (Comp. C), 1 mM AICAR or 30 μM LY294002 (LY) for 1 h before treatment with 50 μM BUT for 6 h. Cell lysates were immunoblotted for the phosphorylation of AMPK, Akt and GSK3β. The columns and errors bars represent means ± SD. ^##^*P* < 0.01 vs control; *P < 0.05, **P < 0.01 vs BUT alone. (**B**) H9c2 cells were transfected with AMPK or scrambled siRNA for 48 h, and then cells were treated with 50 μM BUT for 6 h. Cell lysates were immunoblotted for the phosphorylation of AMPK, Akt and GSK3β. ^##^*P* < 0.01 vs control; ^&&^*P* < 0.01 vs scrambled siRNA.

To examine whether AMPK-mediated GSK-3β phosphorylation need PI3K/Akt phosphorylation, H9c2 cells were treated with BUT with or without pretreatment with LY294002 or Compound C. Both BUT-induced and AICAR-induced Akt and GSK-3β phosphorylation were inhibited by pre-incubation with the AMPK inhibitor, Compound C. Moreover, BUT induced Akt and GSK-3β phosphorylations were inhibited by pre-incubation with LY294002, but did not alter the phosphorylation of AMPK induced by BUT (Fig. 8A). We further confirmed the role of AMPK in BUT-induced Akt/GSK-3β phosphorylations. As shown in Fig. 8B, treatment with AMPK siRNA markedly suppressed the effect of BUT on AMPK, Akt and GSK-3β phosphorylation. Thus, these results indicated that the PI3K/Akt pathway is necessary for BUT-induced AMPK-mediated GSK-3β phosphorylation.

## Discussion

Diabetes is a serious public health problem. Improvements in the treatment of noncardiac complications from diabetes have resulted in heart disease becoming a leading cause of death in diabetic patients^17^. Diabetic cardiomyopathy (DCM), characterized by both early-onset diastolic and late-onset systolic dysfunctions, has been associated with both type 1 and type 2 diabetes^18^. Hyperglycemia, a major etiological component in the development of DCM, is known to promote the production of ROS and reactive nitrogen species (RNS) and/or to deplete antioxidant mechanisms in many cell types, including myocardial cell. It also causes rapid changes in membrane function, followed by contractile dysfunction within weeks in the diabetic heart^19^. Oxidative stress, being an imbalance between endogenous ROS and antioxidant systems in favor of the former, is involved in the etiology of diabetes-induced downregulation of heart function^20^. In the previous study, we found that BUT could protect myocardial I/R in normal rats. But the role of BUT in protecting heart from I/R injury in diabetes and the mechanism is largely unknown. In the present study, we provided direct evidence *in vivo* that BUT protected the heart against I/R injury in diabetic mice as evidenced by significantly improved cardiac function and reduced myocardial apoptosis. We also found that BUT attenuated myocardial injury in diabetic mice possibly through its anti-oxidant effect via activating AMPK/Akt/GSK-3β/Nrf2 signaling.

Using I/R-induced heart injury model in diabetic mice to imitate the DCM has been successfully established, reflected by significantly progressive decreases of cardiac dysfunction, including the decreases of LVDP, +LV dP/dt_max_ and LV dP/dt_min_, along with the significant increases of infarct size and cardiomyocyte necrosis. Pretreatment with BUT could reverse this phenomenon and the effect of 40 mg/kg was as good as metformin. To further investigate whether BUT has direct cardioprotective effects against I/R injury, H9c2 cells were used. BUT increased survival rate, inhibited apoptosis in H9c2 cells subjected to I/R injury, upregulated Bcl-2 protein, downregulated Bax protein and caspase-3 expression in cardiomyocytes.

There are several mechanisms involved in the development of DCM, including increased oxidative stress^20^. In the STZ-induced diabetic rats subjected to I/R injury, hyperglycemia, an independent risk factor, worsens cardiac performance, cell survival, and tissue injury following myocardial I/R via increased oxidant production and reduced antioxidant defenses^21^. Several studies have reported beneficial effects of a therapy with antioxidant agents, including trace elements and other antioxidants, against the cardiovascular system consequences of diabetes^22^,^23^. In the present study, we showed that BUT enhances activities in antioxidant enzymes (GSH-Px, GSH, SOD, CAT and GR) and decreases the levels of MDA and ROS in heart tissues, and further study also showed GSH-Px, GSH, SOD, CAT and HO-1 at both mRNA and protein levels were increased in H9c2 cells. Those results suggested that BUT protect the heart from injury by scavenging free radicals and also improving the endogenous antioxidant system in I/R-treated diabetic mouse.

Our investigations in the underlying mechanisms of the cardioprotective effects of BUT manifested the activation of the Nrf2 and AMPK/Akt/GSK-3β pathways. The Nrf2 pathway was regarded as the most important in the cell to protect against oxidative stress^24^. The antioxidant response element (ARE), a cis-acting enhancer sequence, controls the basal and inducible expression of antioxidant genes in response to antioxidants and UV light^25^. Nrf2 binds to the ARE and regulates ARE-mediated antioxidant enzyme genes expression, including NQO1, HO-1 and other antioxidants^6^. Therefore, it is of particular interest to determine whether BUT can activate Nrf2 in association with HO-1 up-regulation in diabetic heart. In this study, BUT significantly upregulated Nrf2 activation, and such was correlated with a significant upregulation of HO-1 expression in heart issues. In H9c2 cells, the cytoplasm and nuclear levels of Nrf2 were increased by treatment with BUT in a dose-dependent manner. Under normal conditions, Nrf2 is tightly bound to Keap1 and is anchored in the cytoplasm, leading to its ubiquitination and subsequent degradation. Electrophilic agents liberate Nrf2 from the Nrf2-Keap1 cytosolic complex, allowing it to traverse into the nucleus to activate ARE-driven gene expression^26^. Our findings indicated that BUT significantly increased the level of Nrf2 by facilitating dissociation of the Nrf2-Keap1 complex and subsequently allowing the nuclear translocation of Nrf2. In addition, the transfection of Nrf2 siRNA abolished the expression of Nrf2-related proteins and the cytoprotective effect of BUT. Those results demonstrated that BUT mediated its cardioprotective effects against oxidative insults through activation of the Nrf2 pathway.

Previous studies suggested that down-regulation of Nrf2 transcriptional activity is dependent on its redistribution back to the cytosol and degradation^27^. Glycogen synthase kinase 3 beta (GSK-3β), a multifunctional serine/threonine kinase, controls switching off of Nrf2 activation of gene expression. GSK-3β phosphorylates Fyn at unknown threonine residue(s) leading to nuclear localization of Fyn, and then phosphorylates Nrf2 tyrosine 568 resulting in nuclear export of Nrf2, binding with INrf2 and degradation of Nrf2^28^,^29^. Our data *in vivo* displayed that GSK-3β phosphorylation was induced by BUT treatment, as well as decreased levels of phosphorylated Fyn. Our study also revealed that BUT enhanced phosphorylation of GSK-3β at inactive form serine 9 in dose- and time-dependent manners in H9c2 cells. This contention is strengthened by our observation that the nuclear accumulation of Nrf2 and expression of Fyn, P-Fyn and HO-1 by BUT was inhibited by GSK-3β overexpression, but restored by blockade of GSK-3β overexpression with LiCl. These data suggested that GSK-3β dependent Fyn inhibition was indispensable to the role of BUT activating Nrf2 in I/R-treated diabetic mouse.

5′-AMP-activated kinase (AMPK) which was known to play a key role in regulating both glucose and fatty acid homeostasis and controlling whole body energy metabolism, has become one of the strategic cellular targets for the treatment of cardiovascular disease associated with diabetes^30^,^31^. Moreover, glucose uptake and metabolism during myocardial ischemia appear to be affected by impairment of AMPK phosphorylation in the heart^32^. Therefore, it is possible that modulation of AMPK activity in the diabetic heart may improve cardiac function and overcome the increased susceptibility of the diabetic heart to I/R injury. In this study, the cytoprotective effect of BUT against I/R injury depended on AMPK activation, as evidenced by the antagonism of AMPK siRNA transcription on the capacity of BUT to recover cell viability and decrease ROS levels against I/R treatment. The serine survival kinase Akt is activated downstream of phosphatidylinositol 3-kinase (PI3K). The PI3K/Akt pathway plays a critical role in promoting cell survival in the heart, and there is increasingly evidence to indicate cross talk between the Nrf2 and PI3K/Akt pathways in response to oxidative insults^33^,^34^. A downstream effector of Akt, GSK-3β is phosphorylated at Ser 9 by Akt and phosphorylated GSK-3β attenuates MI/R injury^35^. In the present study, I/R decreased Akt phosphorylation in diabetic heart or H9c2 cells and BUT pretreatment augmented Akt phosphorylation significantly. Further study also showed that Akt siRNA transcription abolished the protective effect of BUT. siRNA transcription study also demonstrated that AMPK and Akt were both necessary to the expression of Nrf2 induced by BUT.

To identify whether AMPK activated by BUT was responsible for the phosphorylation of Akt and GSK-3β, H9c2 cells were treated with the selective AMPK inhibitor, Compound C. Pretreatment with Compound C reduced the BUT-stimulated increase in the phosphorylation of AMPK, Akt and GSK-3β. Moreover, pretreatment with the PI3K inhibitor, LY249002, greatly diminished the phosphorylation of Akt and GSK-3β evoked by BUT. However, LY294002 only has little effect on BUT-stimulated AMPK phosphorylation. Thus, BUT not only induced phosphorylation of AMPK but also caused the phosphorylation of Akt and GSK-3β in an AMPK-dependent manner. These results indicated that the AMPK/Akt/GSK3β pathway is responsible for BUT’s pharmacological action.

In summary, this study demonstrated that BUT possesses cardioprotective properties against oxidative injury and myocardium I/R *in vivo* and *in vitro*. The underlying mechanisms of BUT mediated cardioprotection may be attributable to activation and cross-talk between the AMPK/Akt and GSK-3β/Nrf2 signaling pathways. These results supported further investigation of BUT as a promising novel therapeutic agent for diabetic and its complications.

## Materials and Methods

### Materials

Butin was purchased from National Institute for the Control of Pharmaceutical and Biological Products, and the purity being more than 98%. Dulbecco’s Modified Eagle’s Medium (DMEM) and other cell culture supplies were purchased from Gibco (Life Technologies, Grand Island, NY). 3-[4, 5-Dimethylthiazol-2-yl]-2, 5-diphenyltetrazolium bromide (MTT) and 2, 3, 5-triphenyltetrazolium chloride (TTC) were obtained from Sigma (St. Louis, MO). The detection kits of malondialdehyde (MDA), superoxide dismutase (SOD), glutathione (GSH) and glutathione disulfide (GSSG) and caspase 3 were purchased from Nanjing Jiancheng Bioengineering Institute (Nanjing, P.R. China). Monoclonal rabbit anti-mouse P-AMPK, AMPK, P-GSK-3β, GSK-3β, Bcl-2, β-actin, Bax, NQO1, HO-1 and Nrf2 antibodies were purchased from Cell Signaling Technologies, MA, USA. All other chemicals used in this experiment were the purest grade commercially available.

### Cell culture

The H9c2 cell line was purchased from the American Tissue Type Collection (ATCC, VA, USA). Cells were grown in complete medium containing DMEM supplemented with 10% fetal bovine serum, 100 U/ml of penicillin and 100 U/ml of streptomycin, and maintained at 37 °C, in a humidified incubator containing 5% CO_2._ The medium was replaced every 2–3 days, and cells were used for experimental procedures at 80–90% confluence. Cells were pretreated with butin (12.5, 25 and 50 μM) or MET (40 μM) for 6 h, and then subjected to I/R. Ischemia was simulated with ischemia buffer (in mM: KCl 10, CaCl_2_ 1.0, NaCl 98.5, HEPES 20, MgSO_4_ 1.2, sodium lactate 40, pH 6.8, 37 °C) for 3 h and then reperfused for 6 h with DMEM. Ischemia conditions were obtained by equilibrating a small humidified Plexiglas chamber containing with 95% N_2_ and 5% CO_2_. Reperfusion conditions were the normal culture condition.

### Animals and diabetic mouse model

The experimental protocol was approved by the Ethics Committee for Animal Experimentation of the Fourth Military Medical University and was performed according to the Guidelines for Animal Experimentation of the Fourth Military Medical University and the National Institute of Health Guide for the Care and Use of Laboratory Animals (NIH Publications No. 80–23) revised in 1996.

DM was induced in male C57/BL6J mice 8–12 weeks old, weighing 23–25 g, by intraperitoneal injection of streptozotocin (STZ, Sigma, St Louis, MO, USA) at a dose of 50 mg/kg dissolved in 100 mM citrate buffer pH 4.5 for 5 consecutive days. Control animals were injected with the same volume of vehicle. After 4 weeks, blood glucose levels were measured using Bayer’s BREEZE2 meter (Bayer Health Care LLC, Mishawaka, USA) by tail vein blood sampling. Mice with blood glucose levels of >11.1 mM were used for the present study (Supplementary Table 1). Mice were housed under controlled conditions with a 12 h light/dark cycle, a temperature at 25 ± 2 °C, and humidity in 60–70%. The mice were allowed free access to standard rodent diet and tap water. Animals whose hyperglycemia had been successfully induced were randomly assigned to six groups: DM + sham, DM + I/R, BUT (10, 20 and 40 mg/kg) and metformin (MET, 50 mg/kg). Tested drugs were given after 4 weeks of STZ injection by gavage every other day for 15 days.

### Ischemia/reperfusion (I/R) model

After different treatment, diabetic mice were anesthetized by 3% pentobarbital sodium. The chest was opened by a middle thoracotomy, then a 6–0 black silk ligature was placed under the left coronary artery, and the ends of the tie were threaded through a small vinyl tube to form a snare for reversible left coronary artery occlusion. After 30 min of ischemia, reperfusion was established by loosening the snare for 6 h. The loosened suture was left in place and then the blood was collected from the carotid artery for subsequent study; and the hearts were removed for assessing the myocardial infarction size. The sham-operated diabetic mice underwent the same operation except for ligation of the coronary artery (DM + sham).

### Determination of Cardiac Function and Myocardial Infarct Size

At the end of the 6-hour reperfusion period, mice were re-anesthetized and cardiac function was determined by invasive hemodynamic evaluation methods. The left ventricular systolic pressure, left ventricular end-diastolic pressure, first derivative of the left ventricular pressure (+dP/dt_max_ and dP/dt_min_) and heart rate were obtained by use of computer algorithms and an interactive videographics program (Po-Ne-Mah Physiology Platform P3 Plus, Gould Instrument Systems, Valley View, Ohio).

After measuring hemodynamic parameters, the left aortic coronary artery was occluded again and 2 ml of 1% Evans blue dye was injected into the femoral vein to distinguish between perfused and non-perfused sections of the heart. The area at risk was separated from the remaining of the left ventricle and then cut into small pieces and incubated with 1% 2, 3, 5-triphenyl tetrazolium chloride (TTC) phosphate buffer (pH 7.4) for 15 min at 37 °C to visualize the infarct area. The areas stained with Evans blue (area not at risk), TTC (red staining, ischemic but viable myocardium, area at risk, AAR) were measured digitally using Image Pro Plus software (Media Cybernetics, USA). The myocardial infarct size was measured and expressed as a percentage of AAR over total left ventricular.

### Biochemical analysis

The collected blood samples were centrifuged at 3000 g at 4 °C for 20 min. Supernatants were transferred to clean EP tubes and stored at −80 °C. Cardiac muscle tissues or H9c2 cells were homogenized at 4 °C in cold buffer (1.5 mM Tris base-HCl, 1 mM DTT, 0.25 M sucrose, 1 mM MgCl_2_, 1.25 mg/mL pepstatin A, 10 mg/mL leupeptin, 2.5 mg/mL aproptonin, 0.5 mM PMSF, 2.5 mM EDTA, 1 mM EGTA, 0.1 M Na_3_VO_4_, 50 mM NaF, and 2 mM sodium pyrophosphate) and then centrifuged at 3000 g for 20 min at 4 °C. The lactate dehydrogenase (LDH), creatine kinase-MB (CK-MB), superoxide dismutase (SOD), glutathione peroxidase (GSH-Px), GSH, GSSG and MDA levels were determined using commercially available rat ELISA kits as per the manufacturer’s protocol.

### Western blot analysis

For western blot analysis, reperfusion was stopped at 6 h and total proteins were extracted from area at risk zones of the heart. The myocardial tissues were placed in RIPA (Radio-Immunoprecipitation Assay) lysis buffer containing 1% PMSF (phenylmethanesulfonyl fluoride) and 1% protease inhibitor cocktail (Roche Applied Science), homogenized and then centrifuged. The cells were harvested and lysed. Cell lysates were centrifuged at 12,500 g for 20 min at 4 °C, and the supernatant was collected and stored at −80 °C. The protein concentration of each sample (cell or tissue) was determined using a BCA protein assay kit (Nanjing Jiancheng). Equal amounts of protein (50 μg) were separated by SDS-PAGE on 10% gels and electrophoretically transferred to a polyvinylidene fluoride membrane (PVDF). Nonspecific binding was blocked with 5% BSA for 1 h at room temperature. The membranes were incubated with primary antibodies diluted 1:500 overnight at 4 °C and then incubated with the secondary antibody at room temperature for 1 h. The immunoreactive bands were detected using the ECL method. Optical densities of the bands were scanned and quantified image analysis systems (Bio- Rad, USA). β -actin served as an internal control.

### Detection of intracellular reactive oxygen species (ROS)

Intracellular ROS generation was monitored by flow cytometry and using peroxide-sensitive fluorescent probe 2′, 7′-dichlorofluorescein diacetate (DCFH-DA, Molecular Probes) and dihydroethidium (DHE). Briefly, the cells were seeded in 6-well plates, after treatment with appropriate concentrations of test samples, cells were incubated with 5 mM DCFH-DA and 5 mM DHE at 37 °C for 20 min in the dark. Cells were washed immediately and re-suspended in 1× PBS, filtered through nylon mesh and analyzed by flow cytometry. The fluorescence emitted at 525 nm was measured and analyzed using the Cell Quest^TM^ software. Ten thousand cells were examined for each sample. The values were expressed as percent of fluorescence in the control.

### SiRNA transfection assay

Double-stranded siRNA sequences targeting Nrf2, GSK-3β and AMPK mRNAs were obtained from Santa Cruz Biotechnology. The non-specific siRNA (scramble) consisted of a nontargeting used as a control. H9c2 cells were seeded at 5 × 10^4^ cells per well in 6-well plates to achieve 40–60% confluence. Cells were transfected with 10 nmol siRNA or scrambled siRNA using SureFECT transfection reagent according to the manufacturer’s instructions. And then cells were treated with BUT for 6 h, and subjected to I/R. Specific silencing was examined by immunoblotting with cellular extracts after transfection.

### Immunofluorescence

H9c2 cells were cultured in a glass culture chamber and exposed to I/R with or without BUT pretreatment for 6 h. Acetone-fixed cells were blocked with 5% BSA for 1 h at 37 °C and then incubated with anti-Nrf2 at 1:50 overnight at 4 °C. After PBS washing, cells were incubated with secondary antibody (1:200), counterstained with DAPI, and mounted, observed under confocal microscope.

### Statistical analysis

Values are expressed as mean ± SD from at least three different experiments. The groups were compared using one-way ANOVA followed by Tukey’s multiple comparison tests using the statistics module of Graph Pad Prism 5.0. Analysis of covariance was used to test the relationship between AAR and Infarct. A value of P < 0.05 was considered statistically significant.

## Additional Information

**How to cite this article**: Duan, J. *et al*. Protective effect of butin against ischemia/reperfusion-induced myocardial injury in diabetic mice: involvement of the AMPK/GSK-3β/Nrf2 signaling pathway. *Sci. Rep.* **7**, 41491; doi: 10.1038/srep41491 (2017).

**Publisher's note:** Springer Nature remains neutral with regard to jurisdictional claims in published maps and institutional affiliations.

## Supplementary Material

Supplementary Information
