# Supplementary material for: Protective effect of butin against ischemia/reperfusion-induced myocardial injury in diabetic mice: involvement of the AMPK/GSK-3β/Nrf2 signaling pathway
Source: Sci Rep. 2017 Jan 27;7:41491. doi: 10.1038/srep41491 (PMC5269748; doi:10.1038/srep41491)

Protective effect of butin against ischemia/reperfusion-induced myocardial injury in diabetic mice: involvement of the AMPK/GSK-3β/Nrf2 signaling pathway

Jialin Duan[a,b 1], Yue Guan[a1], Fei Mu[a1], Chao Guo[a1], Enhu Zhang[b1], Ying Yin[a], Guo Wei[a], Yanrong Zhu[a], Jia Cui[a], Jinyi Cao[a], Yan Weng[a], Yanhua Wang[a], Miaomiao Xi[a*], Aidong Wen[a*]

[a]Department of Pharmacy, Xijing Hospital, Fourth Military Medical University, Xi'an, 710032, China.

[b]College of Pharmacy, Shaanxi University of Chinese Medicine, XianYang 712083, PR China

[1] These authors equally contributed to this work.

[*]Corresponding authors. Prof. Wen and Prof. Xi both are contacted at Changle West Road 127, Xi'an, Shaanxi, China. Tel.: +86 29 8477 3636; Fax: +86 29 8477 3636.

***Email address*:** aidongwen2015@163.com (AD. Wen), miaomiaoxi2014@163.com (MM. Xi).

Supplemental figures

Supplementary table 1: Effect of BUT on the levels of blood glucose.

Parameter	Control	Model	BUT(10)	BUT(20)	BUT(40)	MET
**Blood glucose (mM)**	7.7±2.4	22.4±3.2$^{\#\#}$	20.3±2.8	16.3±3.3**	13.6±3.1**	13.1±2.7**

Values (n=6–8 per group) are expressed as means ± SD. $^{\#\#}$*P*<0.01 vs control group, ***P*<0.01 vs model group. Control group: normal mice; Model: diabetic mice; BUT(10), BUT(20), BUT(40): diabetic mice were treated with 10, 20 and 40 mg/kg BUT, respectively; MET: diabetic mice were treated with 50 mg/kg MET.

Supplementary table 2: The relationship between the size of AAR and the size of Infarct was plotted and analyzed using analysis of covariance.

Tests of Between-Subjects Effects

Dependent Variable: Infarct

Source	Type III Sum of Squares	df	Mean Square	F	Sig.
Corrected Model	2662.247[a]	8	332.781	70.258	.000
Intercept	.026	1	.026	.005	.942
AAR	13.605	1	13.605	2.872	.111
GROUP	1253.281	7	179.040	37.800	.000
Error	71.048	15	4.737		
Total	6031.069	24			
Corrected Total	2733.295	23			

a. R Squared = .974 (Adjusted R Squared = .960)

Supplementary figure 1: (A) Plasma creatine kinase-MB (CK-MB) and (B) lactate dehydrogenase (LDH) levels. Values (n=6–8 per group) are expressed as means ± SD. ##P<0.01 vs DM+sham group, **P<0.01 vs DM+I/R group, &P<0.05 vs I/R group.

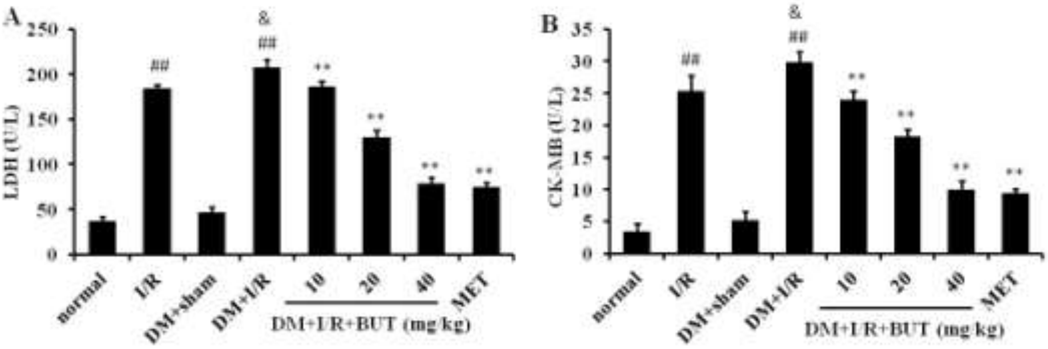

Supplemental figure 2: BUT up-regulated the expression of GSH-Px, GSH, CAT, GR in the heart. Mice received I/R treatment in the presence or absence of BUT pre-treatment, the expression level of GSH-Px (A), GSH (B), CAT (C) and GR (D) in the heart were measured as described in Materials and methods. Values (n=6–8 per group) are expressed as means ± SD. $^{\#\#}P<0.01$ vs DM+ sham group, $^{**}P<0.01$ vs DM+I/R group.

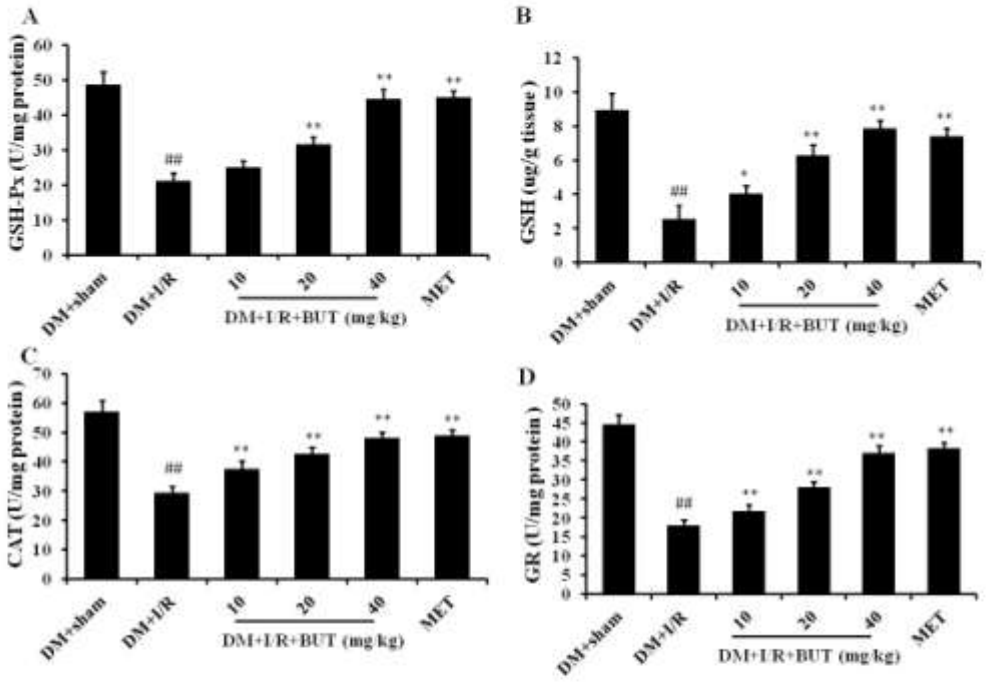

Supplemental figure 3: Effects of BUT on AMPK, Akt and GSK3β phosphorylation and Nrf2 expression in heart treated with I/R. Immunoblotting of protein extracts from heart of mice treated with BUT (40 mg/kg). Levels of Nrf2 expression and AMPK, Akt, GSK3β phosphorylation in the hearts of mice treated with BUT. The columns and errors bars represent means ± SD. $^{\#\#}P$<0.01 vs sham group, $^{**}P$<0.01 vs I/R group.

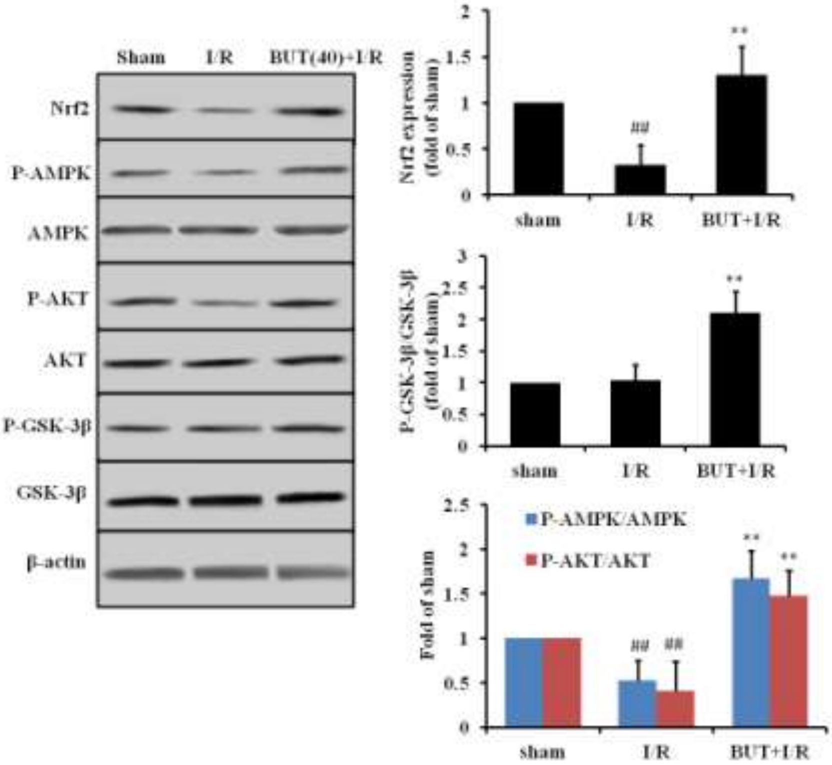

Supplemental figure 4: BUT induced the cytoplasm expression and nuclear translocation of Nrf2 determined by immunofluorescence staining. Left panel: green fluorescence showing Nrf2 localization; Middle panel: stained nucleus with DAPI.

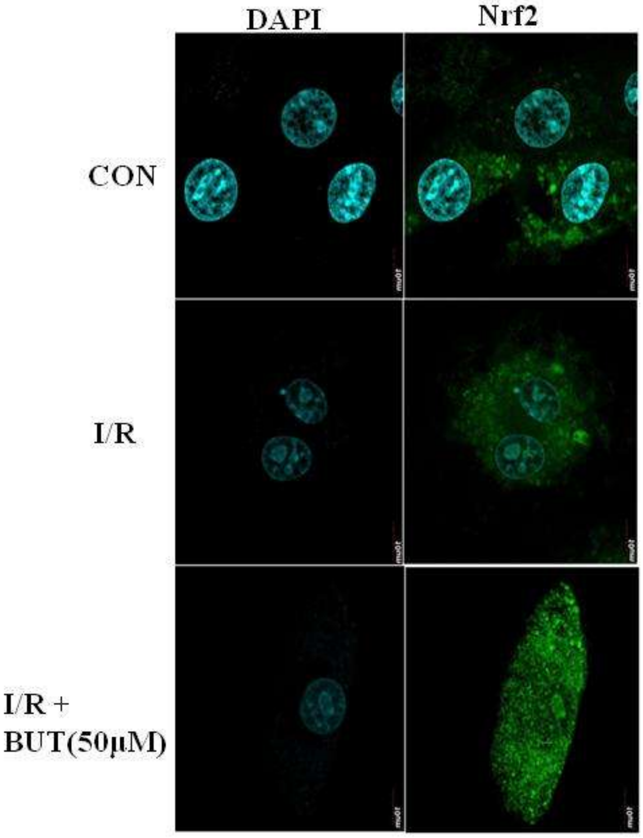

Supplemental figure 5: Cell viability (A) and Ros (B) levels were determined after siRNA transfection. The columns and errors bars represent means ± SD. ***P*<0.01 vs I/R group. ##*P*<0.01 vs control group. &&*P*<0.01 and $$*P*<0.01 vs crambled control RNA.

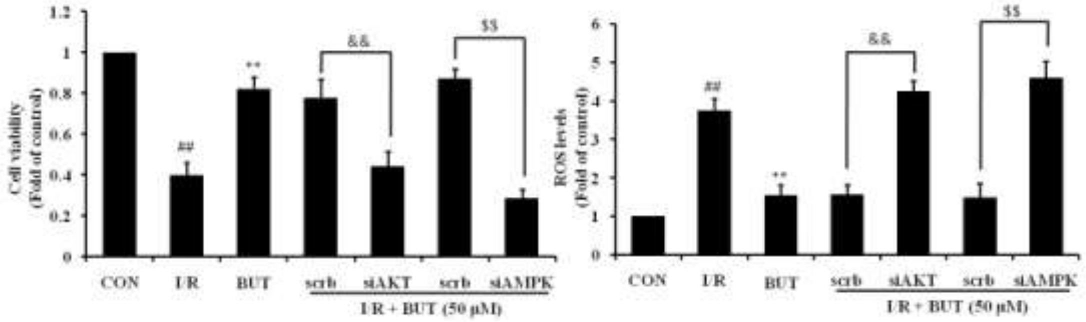

Supplement: Supplementary Information [file srep41491-s1.pdf]
